# Expert Review of the Strategies to Optimize Long-Term Outcomes After Coronary Artery Bypass Grafting

**DOI:** 10.31083/RCM39887

**Published:** 2025-11-27

**Authors:** Shiva Seyed Mokhtassi, Halil Ibrahim Bulut, Yousuf Salmasi, Espeed Khoshbin

**Affiliations:** ^1^Department of Cardiothoracic Surgery, Harefield Hospital, Royal Brompton, and Harefield as Part of Guys and St Thomas NHS Trust, UB9 6JH London, UK; ^2^Department of Cardiothoracic Surgery, St George's University Hospitals NHS Foundation Trust, SW17 0QT London, UK; ^3^National Heart & Lung Institute, Imperial College, SW3 6LY London, UK; ^4^Department of Cardiothoracic Surgery, Hammersmith Hospital as Part of Imperial College Healthcare NHS Trust, W12 0HS London, UK

**Keywords:** coronary artery bypass grafting, secondary prevention, medical management

## Abstract

Coronary artery bypass grafting (CABG) remains a cornerstone in the treatment of advanced ischemic heart disease, offering durable and effective revascularization. Despite surgical success, long-term patient outcomes are often shaped by the progression of native coronary disease and the development of comorbid conditions. This narrative review explores seven critical domains in secondary prevention following CABG: Early recognition of postoperative complications, evidence-based pharmacotherapy, management of atrial fibrillation, lifestyle modification, psychological well-being, preservation of ventricular function, and collaboration within the multidisciplinary team. Effective secondary prevention can significantly reduce the risk of further cardiovascular events and support the longevity of the graft. Interventions such as lipid management, smoking cessation, and structured cardiac rehabilitation promote both physiological recovery and emotional resilience. Timely treatment of arrhythmias and ventricular dysfunction further reduces the risk of heart failure and recurrent ischemia. Primary care practitioners are uniquely positioned to lead the delivery of long-term secondary prevention. By integrating evidence-based strategies into routine care, these strategies can play a pivotal role in improving quality of life and long-term outcomes for post-CABG patients.

## 1. Introduction

**Fig. 1.**
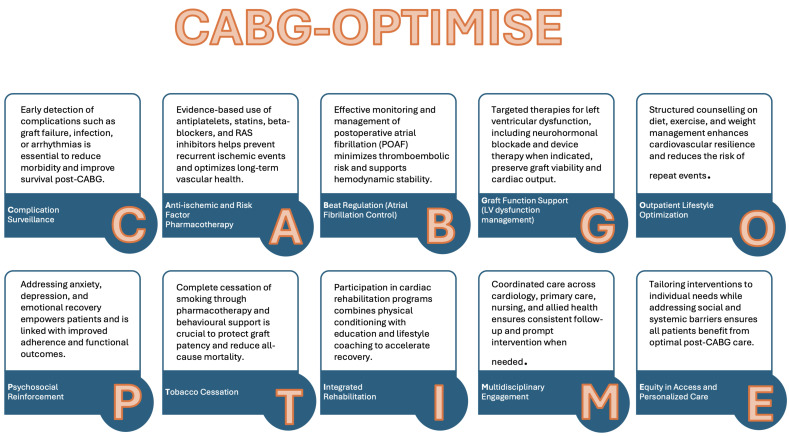
**CABG-OPTIMISE framework: a multidimensional strategy for enhancing long-term outcomes after coronary artery bypass grafting, including primary and multidisciplinary care integration**. CABG, coronary artery bypass grafting; RAS, renin–angiotensin system.

This review consolidates current evidence and evolving perspectives on secondary prevention after coronary artery bypass grafting (CABG). To structure the synthesis, we developed the CABG-OPTIMISE framework (Fig. 1), comprising nine interlinked domains:

C- complication surveillance.

A- anti-ischemic and risk factor pharmacotherapy.

B- beat regulation (atrial fibrillation control).

G- graft function support (left ventricular (LV) dysfunction).

O- outpatient lifestyle optimization.

P- psychosocial reinforcement.

T- tobacco cessation.

I- integrated rehabilitation.

M- multidisciplinary engagement.

This structure aims to provide clinicians with a practical roadmap to enhance long-term outcomes in patients undergoing CABG.

## 2. Methods

This review synthesizes current evidence on secondary prevention strategies to optimize outcomes in patients undergoing CABG.

### 2.1 Search Strategy

A systematic literature search was conducted in PubMed, MEDLINE, Embase, and the Cochrane Library for peer-reviewed articles published between 2000 and 2024. Search terms included “coronary artery bypass grafting”, “secondary prevention”, “risk factor management”, “cardiac rehabilitation”, “lifestyle modifications”, and “post-CABG complications”.

### 2.2 Eligibility Criteria

Studies were included if they:

- Focused on CABG patients.
- Reported on secondary prevention interventions (e.g., pharmacologic management, risk factor control, lifestyle modification, or rehabilitation).
- Were published in English.
- Included randomized trials, cohort studies, systematic reviews, or meta-analyses.

Studies were excluded if they addressed only surgical technique, preoperative care, or lacked clinical outcome data.

### 2.3 Data Extraction and Analysis

Extracted data included study design, patient characteristics, intervention type, outcomes (e.g., graft patency, cardiovascular events, mortality), and follow-up duration. Seven thematic domains were initially examined:

1. Early recognition of postoperative complications.
2. Pharmacological management.
3. Postoperative atrial fibrillation (POAF).
4. Management of left ventricular dysfunction.
5. Lifestyle modifications.
6. Cardiac rehabilitation.
7. Smoking cessation.
8. Psychological wellbeing.
9. Role of the multidisciplinary team.

### 2.4 Quality Assessment

Included studies were evaluated for methodological quality using the Newcastle-Ottawa Scale (for observational studies) and Cochrane risk-of-bias tools (for randomized controlled trials). Only high-quality studies were retained in the final analysis.

**Fig. 2.**
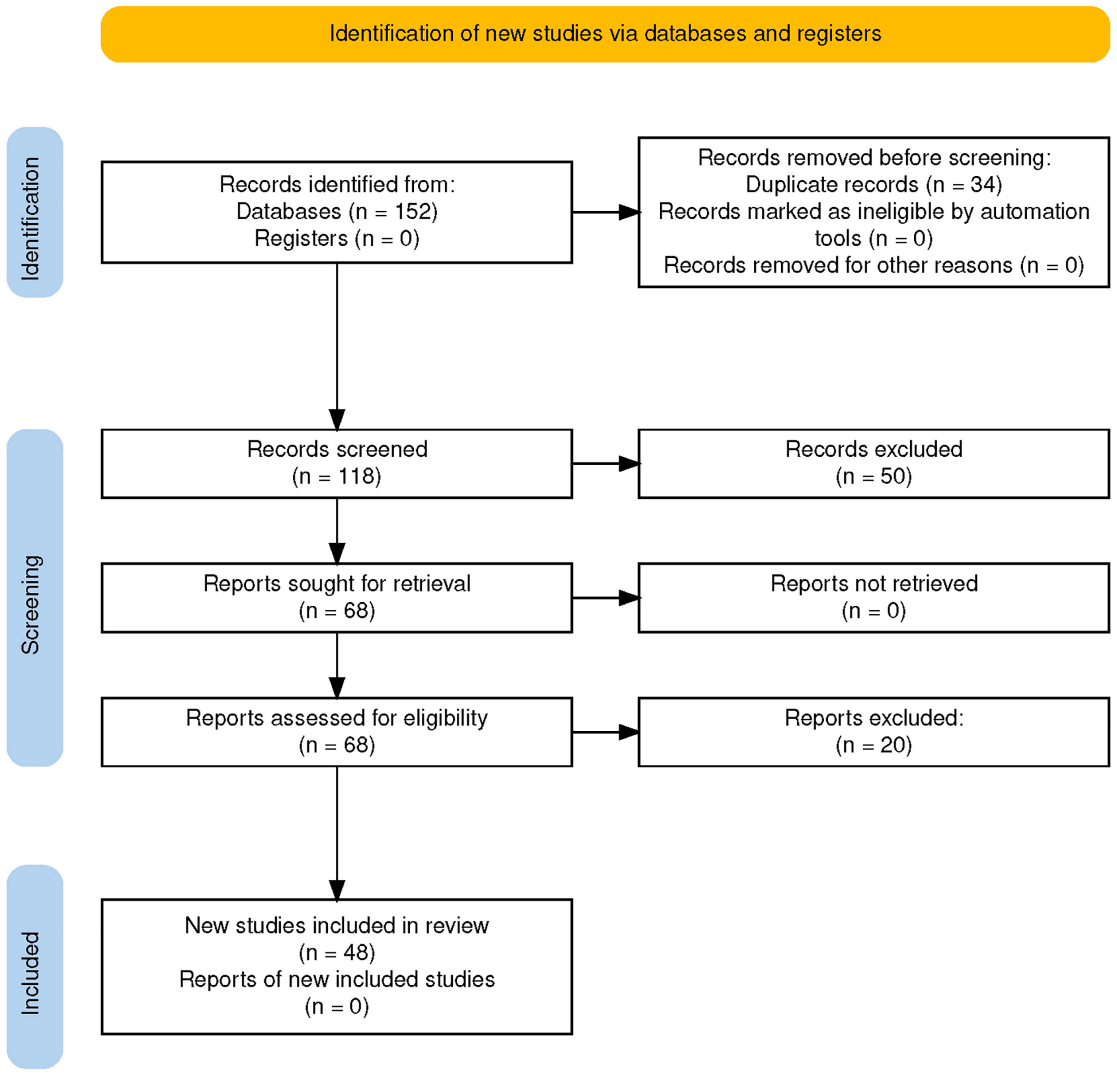
**PRISMA flow diagram illustrating the study selection process for qualitative synthesis**.

A Preferred Reporting Items for Systematic Reviews and Meta-Analyses (PRISMA) flow diagram (Fig. 2) summarizing the selection process is provided below.

## 3. Discussion

### 3.1 Early Recognition of Postoperative Complications

Embedding early complication surveillance into routine post-CABG care improves continuity, reduces readmissions, and enhances long-term outcomes. Common early complications post coronary artery bypass grafting include:

- POAF: Occurs in up to 30% of patients; increases stroke risk and prolongs hospitalization.
- Acute kidney injury (AKI): Often related to hypotension or nephrotoxic agents; associated with higher morbidity.
- Cerebrovascular events: Stroke or transient ischaemic attack (TIA) may arise from embolic or hemodynamic mechanisms.
- Bleeding and tamponade: Require close monitoring of drain output and hemodynamic status.
- Wound infections: Deep sternal infections, particularly in patients with diabetes or obesity, can delay healing and increase mortality.
- Fluid overload: Resulting from intraoperative shifts or impaired cardiac function; may lead to peripheral edema or pulmonary congestion.

Importantly, these early complications should be distinguished from progressive coronary disease or late graft failure due to factors like conduit thrombosis or competitive flow.

Despite their prevalence, early postoperative issues are often under-recognized in clinical practice. Structured follow-up—ideally within 7–14 days post-discharge—is essential for early detection and intervention. This visit should include:

- Wound and rhythm assessment.
- Volume status evaluation.
- Medication review.

New or worsening symptoms (e.g., chest pain, dyspnea, fatigue) should prompt tailored investigations, such as:

- Stress echocardiography or myocardial perfusion imaging—to evaluate ischemia.
- Computed Tomography coronary angiography—for stable patients with suspected graft dysfunction.
- Invasive coronary angiography—when early graft failure is strongly suspected.
- Echocardiography, B-type natriuretic peptide (BNP)/N-terminal pro-B-type natriuretic peptide (NT-proBNP), electrocardiogram (ECG), and troponin—to assess ventricular function and rhythm.

Table 1 summarizes follow-up and diagnostic strategies stratified by clinical risk.

**Table 1.** **Risk-stratified follow-up timing and diagnostic approach after coronary artery bypass grafting (CABG)**.

Patient risk group	Recommended follow-up timing	Suggested diagnostic tools
All patients post-CABG	Within 7–14 days post-discharge	Clinical review, wound check, medication reconciliation, electrocardiogram (ECG)
High-risk for complications (e.g., chronic kidney disease, diabetes, frailty)	Within 7 days post-discharge and again at 30 days	Renal function panel, volume status, wound inspection, ECG
Patients with recurrent symptoms (angina, dyspnea)	Immediate evaluation; no delay in referral	Stress echo, myocardial perfusion imaging, ECG, labs
Patients with suspected graft failure	Within 1 month or sooner if unstable	computed tomography (CT) coronary angiography or invasive coronary angiography
Patients with abnormal ECG or elevated biomarkers	Prompt inpatient or urgent outpatient work-up	High-sensitivity troponin, B-type natriuretic peptide(BNP)/N-terminal pro-B-type natriuretic peptide (NT-proBNP), echocardiogram
Asymptomatic patients	Routine clinical review only; no routine imaging	No testing unless new symptoms or clinical concerns arise

### 3.2 Pharmacological Management

#### 3.2.1 Dual Antiplatelet Therapy

Dual antiplatelet therapy (DAPT) is a cornerstone of secondary prevention after CABG, particularly in patients with acute coronary syndrome (ACS). The choice and duration of antiplatelet therapy are guided by the clinical context of either ACS or chronic coronary syndrome (CCS) [1, 2].

In ACS, guidelines recommend 12 months of DAPT, typically aspirin plus a P2Y₁₂ inhibitor (clopidogrel or ticagrelor), to minimize recurrent thrombotic risk [3]. In CCS, aspirin monotherapy remains standard; however, short-term DAPT (1–6 months) may be beneficial in high-risk cases—especially with saphenous vein grafts (SVGs) [4].

DAPT has been shown to reduce SVG failure rates from 20% to approximately 11.2%, though this comes at the cost of increased bleeding [2]. The DACAB trial reported a 9% absolute reduction in SVG occlusion with ticagrelor-based DAPT, but with elevated bleeding risk [5], highlighting the need for individualized risk-benefit analysis.

Standard DAPT duration in ACS is 12 months, regardless of revascularization strategy [6, 7]. While studies such as found no mortality benefit with extended DAPT beyond one year in stable patients, premature discontinuation—especially within the first 3 months—has been linked to increased graft thrombosis [6, 7].

Emerging evidence supports tailoring DAPT by graft type. SVGs derive more benefit from DAPT compared to arterial grafts, such as the left internal mammary artery (LIMA), which tend to have higher patency and lower thrombosis rates [2].

#### 3.2.2 Anticoagulation 

Routine anticoagulation to enhance graft patency following CABG is not recommended. Current guidelines advise against the use of oral anticoagulants for this indication due to insufficient benefit and elevated bleeding risk, particularly in the early postoperative period [8].

The COMPASS-CABG substudy, a component of the larger COMPASS trial, compared low-dose rivaroxaban (2.5 mg twice daily) plus aspirin versus aspirin monotherapy in stable post-CABG patients. After one year, no significant difference in graft patency was observed on coronary CT angiography, despite broader vascular benefits in the main trial cohort [9]. These findings reinforce that anticoagulation should not be used solely to preserve graft patency in the absence of another clear indication.

Atrial fibrillation (AF)—particularly POAF—is the leading indication for long-term anticoagulation following CABG. POAF is common, especially in older patients and those with structural heart disease, and carries a substantial risk of thromboembolic events.

The 2024 European Society of Cardiology (ESC) guidelines recommend using the CHA₂DS₂-VA score (excluding female sex as a risk factor) to assess stroke risk. A score ≥2 warrants anticoagulation; a score of 1 may justify treatment depending on individual clinical context [10].

For non-valvular AF, non-vitamin K oral anticoagulants (NOACs)—apixaban, rivaroxaban, dabigatran, and edoxaban—are preferred due to favorable safety profiles and ease of administration. Warfarin remains indicated for patients with mechanical heart valves or moderate-to-severe mitral stenosis.

Bleeding risk should be assessed with the HAS-BLED score. A score >2 suggests the need for closer monitoring but is not an absolute contraindication to anticoagulation [10, 11].

Anticoagulation after CABG should be reserved for clear clinical indications—primarily atrial fibrillation—rather than employed routinely to preserve graft patency. A selective, risk-based approach is essential, particularly in light of emerging considerations. Patients with malignancy or antiphospholipid syndrome may require individualized anticoagulation regimens, although data specific to the post-CABG setting remain limited [12, 13]. In cases of sustained atrial arrhythmias or the presence of a ventricular thrombus, early anticoagulation may be warranted. However, decisions must carefully weigh bleeding risk, rhythm status, and the clinical context [14, 15]. Utilizing real-world data and dynamic risk stratification tools—such as CHA₂DS₂-VA and HAS-BLED—can guide anticoagulation decisions throughout the continuum of follow-up [16].

In summary, anticoagulation should be initiated based on sound clinical rationale, with a focus on thromboembolic risk mitigation. A personalized, evidence-based strategy remains central as practice continues to evolve.

#### 3.2.3 Lipid Management

Statins remain the foundation of lipid management after CABG and are supported by the most extensive body of clinical evidence. Large randomized trials have consistently shown that high-intensity statin therapy lowers low-density lipoprotein cholesterol (LDL-C) and substantially reduces cardiovascular events. Current guidelines from the American College of Cardiology (ACC) and American Heart Association (AHA), ESC, ACC/AHA, ESC, and American Association of Clinical Endocrinology (AACE) recommend statins as first-line therapy, with a goal of achieving at least a 50% reduction in LDL-C. If LDL-C levels remain above 70 mg/dL despite maximally tolerated statin therapy, ezetimibe is the next step, and proprotein convertase subtilisin/kexin type 9 (PCSK9) inhibitors may be considered for patients with persistent elevations or documented statin intolerance [17, 18, 19, 20].

PCSK9 inhibitors, such as evolocumab and alirocumab, have demonstrated additional LDL-C reduction and modest reductions in major cardiovascular events in the FOURIER and ODYSSEY OUTCOMES trials. Although these results are encouraging, the breadth and duration of evidence supporting their use remain less robust than for statins. Subgroup analyses suggest that post-CABG patients with recurrent ischemia or graft disease may derive particular benefit from more aggressive lipid lowering [17].

Guideline recommendations are moving toward stricter LDL-C targets, with a threshold of <55 mg/dL (1.4 mmol/L) for very high-risk populations, including CABG recipients. Achieving lower levels has consistently been associated with improved outcomes, and concerns about adverse effects at extremely low concentrations (<30 mg/dL) have not been substantiated in clinical trials. Long-term safety data are still accumulating [17].

Newer non-statin agents expand the therapeutic landscape. Inclisiran, a siRNA therapy targeting hepatic PCSK9 synthesis, offers sustained LDL-C lowering with twice-yearly dosing and may improve adherence in patients managing multiple medications [21, 22]. Bempedoic acid, an oral adenosine triphosphate (ATP) citrate lyase inhibitor, is an alternative for statin-intolerant patients and has a favorable safety profile [21]. Lipoprotein(a) is increasingly recognized as an independent driver of residual risk, especially in patients with early-onset coronary disease or graft atherosclerosis. Pelacarsen, an antisense oligonucleotide under investigation in the HORIZON trial, may offer future options for lowering Lp(a) [23]. In addition, icosapent ethyl, a purified eicosapentaenoic acid (EPA) derivative, reduced ischemic events in the REDUCE-IT trial and may be considered in patients with elevated triglycerides despite adequate LDL-C control [24].

In summary, while several novel therapies are emerging, statins remain the bedrock of lipid management after CABG. A stepwise approach—initiating high-intensity statins, adding ezetimibe, and considering PCSK9 inhibitors or other non-statin agents in selected patients—provides the most practical and evidence-based strategy to improve long-term outcomes and preserve graft function.

#### 3.2.4 Beta-Blockers

Beta-blockers have been a mainstay in the management of ischemic heart disease since the 1980s. By reducing heart rate, myocardial contractility, and sympathetic tone, they lower myocardial oxygen demand and enhance diastolic coronary perfusion [25, 26, 27, 28, 29]. Their antihypertensive effect, primarily via reduced cardiac output, further supports their use in cardiovascular care [25, 28].

In the post-CABG setting, beta-blockers are strongly recommended for patients with prior myocardial infarction or left ventricular dysfunction, in the absence of contraindications. Perioperative initiation, particularly before surgery, has been shown to reduce the incidence of POAF. Commonly used agents include bisoprolol, metoprolol succinate, and carvedilol.

These indications are endorsed by international guidelines and supported by robust evidence [25, 30]. However, caution is necessary in patients with severe bradycardia, hypotension, or reactive airway disease [27, 30].

While beta-blockers remain fundamental in managing patients with reduced left ventricular ejection fraction (LVEF) or high arrhythmic risk, their long-term role in individuals with preserved LVEF is increasingly debated. Emerging evidence indicates minimal long-term advantage of beta-blockers in patients with ischemic heart disease and preserved LVEF. A meta-analysis in *Heart Failure Reviews* found no significant reduction in mortality or hospitalizations in patients with heart failure with preserved ejection fraction (HFpEF) treated with beta-blockers [31].

The 2024 REDUCE-AMI trial, presented at the ACC Scientific Sessions, evaluated post-myocardial infarction (post-MI) patients with LVEF ≥50%. It showed no difference in all-cause mortality or recurrent MI between those treated with beta-blockers and those who were not, challenging the conventional assumption of universal benefit in this cohort [28, 32].

These findings support a more selective approach. While beta-blockers remain essential in patients with reduced LVEF, prior MI, or arrhythmia risk, their routine use in preserved LVEF—particularly beyond the early postoperative or post-MI phase—may not be warranted. Potential side effects, including bradycardia, fatigue, and metabolic disturbances, should be considered when evaluating long-term therapy.

Future guidelines may increasingly emphasize individualized use over blanket prescriptions, aligning beta-blocker therapy with patient-specific risk profiles and evolving evidence.

#### 3.2.5 Antihypertensives

Hypertension is one of the most prevalent comorbidities in CABG patients and a significant contributor to graft failure, recurrent cardiovascular events, and mortality. Effective management is therefore critical to long-term outcomes [33].

In the immediate postoperative period, beta-blockers are often first-line for blood pressure (BP) control and arrhythmia prevention, especially in patients with prior MI or LV dysfunction [26]. Angiotensin converting enzyme (ACE) inhibitors are commonly added for patients with reduced ejection fraction, recent MI, diabetes, or chronic kidney disease (CKD), but should be initiated cautiously with close monitoring of renal function and electrolytes, particularly in those with hemodynamic instability [34].

For patients with heart failure and reduced ejection fraction (HFrEF), angiotensin receptor–neprilysin inhibitors (ARNIs) such as sacubitril/valsartan have demonstrated superiority over ACE inhibitors in reducing cardiovascular mortality and hospitalizations, as shown in the PARADIGM-HF trial [35]. While not typically started in the immediate postoperative phase due to hypotension risk, ARNIs should be considered once patients are clinically stable, especially if LV dysfunction persists [35, 36].

Current guidelines recommend a BP target of <140/85 mmHg, though this is not based on CABG-specific trials [33]. If BP remains uncontrolled on beta-blockers and ACE inhibitors, calcium channel blockers or diuretics may be added. In patients without MI or LV dysfunction, other antihypertensive classes may be favored over beta-blockers, depending on comorbidities and response.

Routine use of ACE inhibitors in the absence of clear indications (e.g., reduced LVEF, recent MI, diabetes, or CKD) is discouraged early postoperatively due to potential hypotension and variable BP responses [34].

Ultimately, post-CABG hypertension management should be individualized, balancing BP control with adequate perfusion and overall patient stability. As further data emerge, personalized approaches will remain essential in this high-risk group.

#### 3.2.6 Sodium-Glucose Cotransporter-2 (SGLT2) Inhibitors 

Agents such as dapagliflozin, empagliflozin, and canagliflozin have demonstrated consistent benefits in HFrEF, independent of diabetic status. Landmark trials (DAPA-HF, EMPEROR-Reduced) showed reductions in heart failure hospitalizations and cardiovascular mortality. Mechanisms include preload reduction, natriuresis, afterload modulation, and enhanced myocardial energetics—making them particularly useful in CABG patients with left ventricular dysfunction [37].

However, SGLT2 inhibitors have shown limited impact on atherosclerotic events, and there is no evidence to suggest improvements in graft patency or progression of native coronary disease post-CABG [37].

#### 3.2.7 Glucagon-Like Peptide-1 (GLP-1) Receptor Agonists

GLP-1 receptor agonists (e.g., liraglutide, semaglutide, dulaglutide) reduce major adverse cardiovascular events (MACE), particularly in patients with type 2 diabetes (T2DM) and obesity, who are at high cardiovascular risk. Trials such as LEADER and SUSTAIN-6 attribute these outcomes mainly to stroke reduction and improved cardiovascular survival, rather than direct effects on myocardial infarction or revascularization rates [38]. Emerging observational data suggest possible additive effects when SGLT2 inhibitors and GLP-1 receptor agonists are used in combination. However, these findings lack validation in surgical cohorts. In post-CABG patients, initiation should be delayed until patients are hemodynamically stable, renal function is preserved, and oral intake is adequate. Therapy selection should reflect comorbidity profiles: SGLT2 inhibitors are preferred for those with HFrEF or CKD. GLP-1 receptor agonists are favored in patients with T2DM and obesity [39].

While these agents represent major advances in cardiometabolic disease management, their precise role in CABG-specific pathways remains under investigation. Future trials will help clarify optimal use in the surgical population.

### 3.3 Management of Postoperative Atrial Fibrillation 

Atrial fibrillation occurs in up to 30% of patients following CABG and is associated with increased risks of stroke, heart failure, prolonged hospital stays, and higher healthcare costs [40]. Management requires a proactive approach that includes prevention, prompt treatment, and long-term planning.

Prevention begins with risk stratification and early intervention. Beta-blockers remain first-line for POAF prophylaxis and should be initiated preoperatively and continued postoperatively, unless contraindicated [26]. In high-risk patients, short-term amiodarone has also been shown to reduce POAF incidence [40].

Electrolyte correction—especially of magnesium—is a simple but crucial step often overlooked in POAF prevention [41]. Increasing evidence also supports the role of inflammation in POAF pathogenesis, prompting interest in anti-inflammatory therapies. Trials such as COPPS and COP-AF have shown that short-term postoperative colchicine use can significantly reduce POAF rates without major side effects [42]. Preoperative high-dose statins (e.g., atorvastatin) may offer additional anti-inflammatory protection. Some studies suggest reduced POAF incidence, although findings remain mixed [43]. Elevated preoperative levels of C-reactive protein (CRP), IL-6, and BNP may identify patients at greater risk for POAF, allowing for more targeted preventive strategies [44, 45].

In patients with atrial fibrillation undergoing CABG, surgical occlusion of the left atrial appendage (LAA) may be considered intraoperatively. The LAAOS III trial demonstrated a significant reduction in ischemic stroke risk when LAA occlusion was performed during cardiac surgery [46, 47]. Although data specific to isolated CABG are limited, this approach may benefit high-risk patients who are unsuitable for long-term anticoagulation or have concerns about medication adherence [47].

The management of POAF is guided by hemodynamic stability and symptom severity. Rate Control is the initial strategy in stable patients. Beta-blockers (e.g., bisoprolol) are preferred. Calcium channel blockers (e.g., diltiazem, verapamil) are alternatives in patients with preserved LV function. Digoxin may be appropriate in hypotensive patients or those with reduced ejection fraction [48]. Rhythm Control should be pursued in hemodynamically unstable patients or if symptoms persist despite adequate rate control. Options include: Amiodarone, Ibutilide, Electrical cardioversion [48].

Anticoagulation is indicated for episodes lasting >48 hours or in patients with elevated CHA₂DS₂-VASc scores, to mitigate stroke risk [48].

In patients with ongoing symptoms or recurrent episodes of POAF, long-term rhythm control may be necessary. Management may include antiarrhythmic medications or catheter ablation, particularly in those with symptomatic, drug-refractory atrial fibrillation. It is essential to address underlying contributing factors such as infection, electrolyte imbalances, hypoxia, or poorly controlled heart failure [49, 50].

Posterior Pericardiotomy is a surgical technique performed during CABG that facilitates pericardial drainage. Randomized trials have shown a significant reduction in POAF rates, likely due to decreased local inflammation [51]. Experimental methods such as vagal nerve stimulation and botulinum toxin injection into cardiac fat pads are under investigation. These approaches aim to modulate autonomic tone and have shown early promise in reducing POAF incidence [52, 53].

In-hospital continuous ECG monitoring is essential for all post-CABG patients. For those presenting with symptoms after discharge, ambulatory monitoring—via Holter monitors, event recorders, or implantable loop recorders—can help detect silent or intermittent POAF, guiding further management [54]. Artificial intelligence–enhanced wearables and ECG platforms are under development to identify asymptomatic POAF earlier and more accurately. These technologies may soon enable more timely and personalized interventions [55, 56].

Following stabilization, long-term care involves reassessing anticoagulation needs based on recurrence risk and promoting comprehensive lifestyle management—including smoking cessation, moderation of alcohol intake, weight control, and optimal management of hypertension and diabetes [57]. Although POAF typically peaks between days 2 and 4 postoperatively and resolves within six weeks in many cases, it warrants careful follow-up. A proactive, structured approach—beginning preoperatively and extending into recovery—reduces complications and supports optimal outcomes [57].

### 3.4 Managing Left Ventricular Dysfunction 

LV dysfunction is common in patients undergoing CABG, often as a consequence of chronic ischemic heart disease [25]. While revascularization typically enhances myocardial perfusion, some patients—particularly those with pre-existing systolic dysfunction or comorbidities such as CKD—may experience persistent or worsening LV function postoperatively [58, 59]. Contributing factors include perioperative volume shifts and fluid overload, underscoring the need for vigilant monitoring and evidence-based therapy.

Guideline-directed medical therapy (GDMT) remains the cornerstone of post-CABG management in patients with HFrEF. For those with LVEF <40%, particularly with prior myocardial infarction or symptomatic heart failure, the following are recommended [58, 59]: Beta-blockers, ACE inhibitors or angiotensin II receptor blockers (ARBs), though increasingly replaced by ARNIs.

The PARADIGM-HF trial demonstrated a 20% reduction in cardiovascular mortality with sacubitril/valsartan compared to enalapril in New York Heart Association classification (NYHA) class II–III patients, supporting its role as first-line therapy in eligible individuals [35]. Mineralocorticoid receptor antagonists (MRAs)—such as spironolactone or eplerenone—are also recommended for patients with ejection fraction (EF) <35% and NYHA class II–IV symptoms to further reduce mortality and hospitalizations [60, 61].

Although direct data in CABG patients are limited, the physiological basis for neprilysin inhibition—enhancing natriuretic peptide activity and reverse remodeling—supports early post-CABG use in stable patients with persistent LV dysfunction. Close monitoring of renal function and blood pressure is essential during initiation [35].

Dapagliflozin and empagliflozin have demonstrated significant benefits in HFrEF, regardless of diabetic status. Their use post-CABG is supported by biologic plausibility and early real-world data, though further studies are needed to define timing and patient selection [62].

In patients with EF <35%, sinus rhythm, and left bundle branch block (QRS ≥150 ms), cardiac resynchronization therapy (CRT) can improve symptoms and promote reverse remodeling. Early postoperative identification of CRT candidates maximizes benefit [63]. BNP, NT-proBNP, and emerging markers like suppression of tumorigenicity 2 (ST2) and galectin-3 can differentiate between transient postoperative myocardial stunning and persistent LV dysfunction. This facilitates earlier escalation of heart failure therapy and tailored management [64, 65].

Table 2 provides a comparative overview of the principal therapeutic strategies for managing left ventricular dysfunction following CABG, including pharmacological agents such as ACE inhibitors, ARBs, β-blockers, mineralocorticoid receptor antagonists, and emerging therapies like ARNI and SGLT2 inhibitors, as well as device-based interventions. This summary highlights the evidence base and relative benefits of each modality in improving survival, reducing hospitalizations, and optimizing cardiac remodeling in the post-CABG setting.

**Table 2.** **Comparative overview of therapies for managing left ventricular dysfunction after CABG**.

Therapy	Indication	Mechanism of action	Key trials/evidence	Clinical considerations
Beta-blockers	Left ventricular ejection fraction (LVEF) <40%, post-MI, symptomatic Heart Failure with Reduced Ejection Fraction (HFrEF)	Decreases heart rate (HR) and oxygen demand; antiarrhythmic	MERIT-HF, CIBIS-II	Initiate in euvolemic state; avoid in acute decompensation
Angiotensin-converting enzyme inhibitor/Angiotensin II receptor blocker	LVEF <40%, post-myocardial infarction (MI), ACEi if ARNI not tolerated	Renin–Angiotensin–Aldosterone System (RAAS) inhibition; decreases afterload/remodeling	SOLVD, VALIANT	Monitor renal function and potassium; avoid dual RAAS use
(ACEi/ARB)				
Angiotensin receptor–neprilysin inhibitor	NYHA II–III, LVEF <40%	Neprilysin + RAAS inhibition	PARADIGM-HF	Superior to ACEi; 36-hr washout from ACEi; monitor for hypotension, hyperkalemia
(ARNI)				
(sacubitril/valsartan)				
Mineralocorticoid receptor antagonist	LVEF <35%, NYHA II–IV	Aldosterone blockade; reduces fibrosis	RALES, textitASIS-HF	Avoid in hyperkalemia or severe chronic kidney disease (CKD); monitor K+ and creatinine
MRA (spironolactone, eplerenone)				
Sodium-glucose Cotransporter-2	LVEF <40%, with or without diabetes	Reduces preload/afterload; improves energetics	DAPA-HF, EMPEROR-Reduced	Caution in hypovolemia; monitor renal function and risk of diabetic ketoacidosis (DKA)
(SGLT2) inhibitors				
Cardiac resynchronization therapy	LVEF <35%, QRS ≥150 ms, Left Bundle Branch Block (LBBB), sinus rhythm	Improves electrical synchrony and contraction	MADIT-CRT, COMPANION	Evaluate ≥3 months post-op; optimize guideline-directed medical therapy (GDMT) first
(CRT)				
Implantable cardioverter-defibrillator (ICD)	Persistent LVEF <35% ≥3 months post-coronary artery bypass grafting (CABG)	Prevents sudden cardiac death	SCD-HeFT, MADIT-II	Not indicated <3 months post-CABG; reassess left ventricular (LV) function
Biomarker-guided therapy	Persistent symptoms; therapy titration	Uses B-type natriuretic peptide (BNP), N-terminal pro–B-type natriuretic peptide (NT-proBNP), suppression of tumorigenicity 2 (ST2), galectin-3 to guide therapy	GUIDE-IT, ongoing studies	Adjunctive tool; interpret in clinical context

### 3.5 Lifestyle Modifications 

Obesity and metabolic syndrome are key modifiable risk factors for poor outcomes after CABG. In many high-income countries, including the U.S., obesity now surpasses smoking as the leading cause of preventable cardiovascular death [66]. Metabolic syndrome—defined by hypertension, dyslipidemia, insulin resistance, and central obesity—is highly prevalent in this group and frequently coexists with type 2 diabetes, amplifying cardiovascular risk.

Postoperative weight management is central to secondary prevention. The American Heart Association (AHA) provides a Class I recommendation for weight control post-CABG [25, 67]. Targets include: body mass index (BMI) between 18.5–24.9 kg/m^2^, waist circumference <89 cm (women) or <102 cm (men). These thresholds are associated with lower cardiovascular event rates [68].

Structured weight loss interventions should be offered to all overweight or obese patients. In those with BMI >35 kg/m^2^ who do not respond to lifestyle therapy, bariatric surgery may be considered (Class IIb recommendation, Level C) following thorough assessment [67]. For patients with normal BMI but suspected visceral adiposity, waist-to-hip ratio is a valuable tool for risk stratification (Class IIa recommendation) [66]. Wearables and mobile apps are increasingly used to monitor diet, activity, and weight in real time. Early evidence suggests they improve adherence to lifestyle changes post-CABG [69, 70].

Initially developed for glycemic control, GLP-1 Receptor Agonists, semaglutide and liraglutide have demonstrated significant weight loss benefits, even in non-diabetic individuals. These agents may serve as adjuncts in high-risk, obese post-CABG patients [71].

Specialist weight management services—combining input from dietitians, physiotherapists, and behavioral health teams—can improve metabolic outcomes, particularly in patients with severe or refractory obesity [72].

### 3.6 Cardiac Rehabilitation

Cardiac rehabilitation (CR) is a cornerstone of recovery following CABG, providing structured support that extends beyond physical reconditioning. It facilitates myocardial recovery, enhances hemodynamic stability, and helps patients adjust to changes in systemic and pulmonary circulation [73].

Early initiation—even during the intensive care unit (ICU) stay—has been associated with faster recovery, fewer complications, and shorter hospitalisation [74]. Whenever feasible, rehabilitation should begin during the inpatient phase and continue seamlessly into outpatient care.

Core components of CR include supervised, individualised exercise therapy tailored to medical status, comorbidities, and functional baseline. Patients should be routinely referred to a CR programme prior to discharge or during early follow-up (usually at 4–6 weeks postoperatively) [73].

For selected low-risk patients, home-based cardiac rehabilitation (HBCR) is a validated alternative to centre-based models. Randomised trials have shown similar improvements in physical capacity, adherence, and patient satisfaction, making HBCR especially valuable in patients facing geographic or mobility constraints [75].

Exercise regimens begin at low intensity, progressing gradually in duration and workload. Long-term goals align with international guidelines, targeting at least 150 minutes of moderate-intensity aerobic activity per week [76]. Beyond physical gains, CR participation is strongly associated with reduced hospital readmissions, lower all-cause mortality, and fewer MACE, reinforcing its role in long-term secondary prevention.

Contemporary CR increasingly incorporates hybrid formats, blending in-person assessments with remote coaching and digital platforms. These scalable models improve access while maintaining clinical efficacy [75]. Devices such as fitness trackers and smartwatches enable real-time monitoring of physical activity, heart rate, and rhythm. They facilitate personalised adjustments and foster greater patient engagement [77].

Modern CR includes nutrition counselling, psychological support, and stress management. These elements are integral to promoting behavioural change, addressing modifiable risk factors, and improving long-term adherence [78].

### 3.7 Smoking Cessation

Smoking remains one of the most potent modifiable risk factors for cardiovascular disease, with an impact on long-term outcomes comparable to diabetes. Beyond its role in macrovascular disease, it promotes endothelial dysfunction and microvascular injury, accelerating atherosclerosis and impairing surgical recovery [79, 80].

In the context of CABG, active smoking significantly increases the risk of graft failure, MACE, and repeat revascularisation. As such, complete cessation is strongly endorsed by all major cardiac guidelines to enhance both early recovery and long-term survival [79, 80, 81].

Cessation support should begin during hospitalisation and continue through follow-up. A comprehensive approach combines behavioural and pharmacological strategies [82]:

- Counselling (individual or group).
- Pharmacotherapy, including:

1. Nicotine replacement therapy (NRT).
2. Bupropion.
3. Varenicline.

- Referral to specialist smoking cessation services when available.

Tobacco use should be reassessed at every clinical encounter, with positive reinforcement and documentation. Patients should also be counselled to avoid second-hand smoke exposure in all settings [83]. Initiating pharmacotherapy in hospital and continuing post-discharge improves quit rates, especially when tailored to individual preferences, prior cessation attempts, and comorbidities [82].

Though not first-line, nicotine-containing e-cigarettes may offer a harm reduction pathway in heavily dependent smokers unresponsive to standard therapies. A preliminary study shows potential, but long-term safety remains under investigation [84].

Mobile applications, web platforms, and text services (e.g., QuitNow, SmokeFree) have demonstrated effectiveness in boosting motivation, adherence, and quit rates by providing accessible, on-demand support [85].

Emerging research into nicotine metabolism variability suggests future potential for personalised cessation therapy, aligning pharmacological interventions with genetic profiles to maximise effectiveness [86].

### 3.8 Psychological Wellbeing

Depression is highly prevalent after CABG, with rates significantly exceeding those in the general population [87]. Beyond affecting quality of life, postoperative depression is associated with poorer medication adherence, reduced engagement in lifestyle modifications, and lower attendance at follow-up—all of which negatively impact recovery and long-term outcomes [87].

Routine screening for depressive symptoms is strongly recommended during the postoperative period and should be integrated with input from both primary care and mental health services [88]. Early identification of psychological distress enables timely intervention, thereby enhancing patient adherence to secondary prevention measures.

Cognitive behavioural therapy (CBT) and collaborative care models have shown clear benefit in this population, improving mood, coping mechanisms, and psychological resilience [89]. While the direct impact of treating depression on cardiovascular outcomes is still under investigation, its effect on recovery, quality of life, and healthcare engagement firmly supports the inclusion of psychosocial support in post-CABG care [89]. Structured CBT programs, in particular, have demonstrated efficacy in facilitating emotional recovery and functional restoration.

Embedding mental health professionals into cardiac rehabilitation teams improves access to care and reduces stigma. This model enhances continuity and fosters a more holistic recovery environment [90]. Web- and app-based CBT tools (e.g., MoodGym, SilverCloud) offer accessible and cost-effective care for patients with mobility limitations or geographic barriers, broadening the reach of psychological support [91].

Recent research underscores the predictive value of psychosocial factors—such as perceived stress, loneliness, and social isolation—in shaping recovery trajectories. Integrating these into traditional risk stratification tools may better guide tailored interventions in the CABG population [92].

### 3.9 The Multidisciplinary Team 

Contemporary revascularisation guidelines advocate for a collaborative “Heart Team” approach in managing complex coronary artery disease, with a Class I recommendation for its use in CABG [19]. While initially assembled to guide revascularisation decisions, the Heart Team’s utility extends well into the postoperative period.

A structured post-CABG multidisciplinary team typically comprising cardiologists, surgeons, primary care physicians, cardiac rehabilitation specialists, pharmacists, dietitians, nurses, and mental health professionals—plays a pivotal role in delivering coordinated, individualised care. Key functions include:

- Medication optimisation and reconciliation.
- Management of postoperative complications.
- Structuring of rehabilitation programmes.
- Risk factor modification.
- Psychosocial support and community reintegration.

Evidence supports the value of regular or ad hoc multidisciplinary team (MDT) meetings for complex or deteriorating cases, improving clinical decision-making and expediting appropriate interventions [93].

By integrating medical, behavioural, and lifestyle domains, the MDT ensures a comprehensive approach that adapts to evolving patient needs throughout recovery.

Secure, cloud-based platforms now facilitate real-time, multi-site collaboration—enhancing continuity, particularly across tertiary and community interfaces [94].

Joint ward rounds involving surgery, cardiology, nursing, and pharmacy have demonstrated benefits in reducing discharge delays, improving communication, and enhancing patient engagement [94].

Emerging models that involve patients and carers in MDT discussions are promoting shared decision-making and improving adherence and satisfaction [94].

## 4. Conclusion

The long-term success of CABG hinges not only on surgical excellence but also on the quality of postoperative care. Beyond the immediate perioperative period, outcomes are increasingly shaped by sustained secondary prevention, evidence-based pharmacotherapy, lifestyle optimisation, and psychosocial support.

A multidisciplinary, patient-centred approach is essential to reduce complications, prevent disease progression, and enhance long-term quality of life. Effective follow-up requires seamless coordination between cardiologists, surgeons, primary care providers, and rehabilitation teams. Structured surveillance and early identification of risk factors are key to improving outcomes.

Primary care physicians, situated at the nexus of chronic disease management, are central to this continuum. Their role in reinforcing risk factor control, facilitating timely referrals, and ensuring continuity of care cannot be overstated. Close collaboration between hospital-based services and community providers mitigates fragmentation and sustains momentum post-discharge.

As new therapies and digital innovations emerge, the opportunity to personalise post-CABG care grows. The goal is not merely to prolong life, but to restore function, promote autonomy, and ensure patients thrive well beyond surgery.
